# Prophylactic inhibition of NF-κB expression in microglia leads to attenuation of hypoxic ischemic injury of the immature brain

**DOI:** 10.1186/s12974-020-02031-9

**Published:** 2020-12-01

**Authors:** Nahla Zaghloul, Dalibor Kurepa, Mohammad Y. Bader, Nadia Nagy, Mohamed N. Ahmed

**Affiliations:** 1 Department of Pediatrics, Division of Neonatology, University of Arizona, 1501 N. Campbell Avenue, Tucson, AZ USA; 2 Department of Pediatrics, Division of Neonatology, Feinstein Institute for Medical Research, Manhasset, NY USA

**Keywords:** Periventricular leukomalacia, White matter brain injury, Cerebral palsy, Hypoxia ischemia, NF-κB, Microglia, Oligodendroglia, Neuroprotection

## Abstract

**Background:**

Periventricular leukomalacia (PVL), a devastating brain injury affecting premature infants, is the most common cause of cerebral palsy. PVL is caused by hypoxia ischemia (HI) and is characterized by white matter necrotic lesions, microglial activation, upregulation of NF-κB, and neuronal death. The microglia is the main cell involved in PVL pathogenesis. The goal of this study was to investigate the role of microglial NF-κB activity and its prophylactic inhibition in a neonate mouse model of HI.

**Methods:**

Transgenic mice with specific knockout NF-κB in microglia and colony stimulating factor 1 receptor Cre with floxed IKKβ (CSF-1R Cre + IKKβ^flox/wt^ ) were used. Postnatal day 5 (P5) mice underwent sham or bilateral temporary carotid artery ligation followed by hypoxia. After HI insult, inflammatory cytokines, volumetric MRI, histopathology, and immunohistochemistry for oligodendroglia and microglial activation markers were analyzed. Long-term neurobehavioral assessment, including grip strength, rotarod, and open field testing, was performed at P60.

**Results:**

We demonstrate that selective inhibition of NF-κB in microglia decreases HI-induced brain injury by decreasing microglial activation, proinflammatory cytokines, and nitrative stress. Rescue of oligodendroglia is evidenced by immunohistochemistry, decreased ventriculomegaly on MRI, and histopathology. This selective inhibition leads to attenuation of paresis, incoordination, and improved grip strength, gait, and locomotion.

**Conclusion:**

We conclude that NF-κb activation in microglia plays a major role in the pathogenesis of hypoxic ischemic injury of the immature brain, and its prophylactic inhibition offers significant neuroprotection. Using a specific inhibitor of microglial NF-κB may offer a new prophylactic or therapeutic alternative in preterm infants affected by HI and possibly other neurological diseases in which microglial activation plays a role.

## Background

Periventricular leukomalacia (PVL) is a major neuropathologic white matter brain injury and the most common cause of cerebral palsy (CP) in premature infants. In the USA, about 65,000 very low birth weight infants (< 1500 g) are born annually. Ten percent of those infants show signs of CP and 25–50% display cognitive or behavioral deficits [1]. In extremely low birth weight infants (birth weight < 1000 g), the incidence of CP is estimated to be approximately 20% [1]. Risk factors implicated in the development of PVL include prematurity associated with immature cerebrovascular development, hypoxic ischemic insults (HI) with lack of appropriate autoregulation of cerebral blood flow, free radical production, energy deprivation, and chorioamnionitis. Affected infants show definitive signs of cerebral palsy such as spastic diplegia or seizures, mental retardation, visual and hearing impairment, scoliosis, or incontinence by 6–9 months of age.

PVL pathology is characterized by focal necrosis, microgliosis, and a decrease in pre-myelinating oligodendrocyte precursors, along with an arrest of differentiation, leading to hypomyelination and ventriculomegaly. Candidate pathological mechanisms that drive oligodendroglial death and white matter injury include oxidative stress, excitotoxicity, and inflammation [2–4]. In neonate rat models of PVL, microglia exhibit a robust and transient response to white matter injury [5]. Microglial density increases rapidly within 24 h after hypoxia ischemia (HI) and continues to rise until 96 h post-HI. Since microglial activation coincides with white matter cell death following HI, we sought to determine whether inhibiting microglial activation is neuroprotective in a neonate mouse model of PVL.

The classical NF-κB signaling is a major regulator of microglial inflammation. Additionally, NF-κB regulates gene expression of proinflammatory cytokines, chemokines, enzymes, and adhesion molecules, many of which are upregulated in PVL [6]. In rodents, NF-κB is rapidly activated following HI [7]. Pharmacological inhibition of NF-κB after HI significantly reduced brain injury and long-term motor and cognitive impairments [7–9]. While these studies implicate NF-κB in the pathogenesis of PVL, the specific cell lineages responsible for NF-κB-mediated cell death remain unknown.

Our data demonstrate that microglial activation by NF-κB is a novel, cell-specific target for PVL pathogenesis. In this study, we demonstrate that prophylactic selective inhibition of NF-κB in the microglia dramatically attenuates HI-induced white matter injury in a mouse model of hypoxic ischemic injury of the immature brain. Inhibition of NF-κB in microglia protected mice from hypomyelination and ventriculomegaly by decreasing proinflammatory mediators and sparing of myelin.

## Materials and methods

### Animals

All procedures were performed in accordance with the NIH Guidelines on the care and use of vertebrate animals and approved by the Institutional Animal Care and Use Committee of the Feinstein Institutes for Medical Research and University of Arizona. Animals were housed in a 12-h light/dark cycle in a virus/Ag-free facility with controlled temperature and humidity and provided with water and food ad libitum. Colony stimulating factor receptor 1 Cre IKKβ^flox/wt^ (CSF-1R-icre; IKKβ^flox/wt^) were generated by breeding C57BL/6 CSF-1R-cre mice (Jackson Laboratories) to IKKβ^flox/flox^ mice (Jackson Laboratories). Cre specificity was confirmed by crossing cre lines to C57BL/6 Rosa26-Stop^Flox^-CAG-tdTomato (Jackson Laboratories) mice and assessed for tdTomato expression by immunohistochemistry. Equal number of male and female mice was used for the studies.

### Genotypes of used animals

Genotypes for all studied pups were determined by qualitative PCR using the following primers:

**Table:** 

Gene	Forward primer (5′-3′)	Reverse primer (5′-3′)
IKKβ	GTC ATT TCC ACA GCC CTG TGA	CCT TGT CCT ATA GAA GCA CAA
iCre+	CAGGGCCTTCTCCACACCAGC	CTGGCTGTGAAGACCATC
Cre−	GGACATGTTCAGGGATCGCCAGGCG	CGACGATGAAAGCATGTTTAGCTG
tdTomato+	CTG TTC CTG TAC GGC ATG G	GGC ATT AAA GCA GCG TAT CC
tdTomato−	AAG GGA GCT GCA GTG GAG TA	CCG AAA ATC TGT GGG AAG TC

### Hypoxia ischemia insult

For our study, we used our animal model of HI injury of the immature brain which was described in detail before [10]. In brief, under complete aseptic precautions, postnatal day 5 (P5) mouse pups were anaesthetized with isoflurane, midline neck incision was performed, and both carotid arteries were temporary ligated (using 6.0 silk sutures double knot) for 10 min. After that, sutures were removed and the neck incision closed. Pups were allowed to recover for 30 min on thermal blanket, then were placed in hypoxia chamber (FiO_2_ 8%) for 20 min after which they returned to their dams. During surgery and recovery and in the hypoxic chamber, pups were placed on thermal blanket and their rectal temperature maintained at 36.5 °C measured by an ultrathin rectal probe (BiosebLab, France). Sham controls at P5 were anaesthetized with isoflurane, and midline neck incision was performed. Both carotid arteries were isolated but not ligated. Then, the neck incision was closed. During surgery and recovery, sham mice were placed on thermal mattress and rectal temperature was maintained at 36.5 °C.

### Histopathological exam

Brain tissue was fixed on P15 in 4% paraformaldehyde for 24 h, processed, embedded in paraffin, and subsequently cut into 6-μm-thick sections. Following deparaffinization, hematoxylin and eosin (H&E) staining was performed according to standard protocols. Typical sections of hippocampus and cerebrum were made in each group of animals.

### Immunohistochemistry

Animals were deeply anesthetized on P15 with a lethal dose of xylazene/ketamine and perfused transcardially with normal saline, then 4% paraformaldehyde. Brains were sectioned coronally at 6-μm-thick using a microtome. Sections were incubated for 2 h at room temperature in TBS+ 1% Triton-X + 10% donkey serum. Samples were incubated for 24 h at 4 °C with primary antibodies, followed by 2-h incubation at RT with secondary antibodies. All images were captured on a Zeiss confocal microscope (Carl Zeiss, Thornwood, NY, USA). The following primary antibodies were used to detect the following markers: tomato lectin (Vector laboratories, Burlingame, CA, USA), CD 68 (AbS Serotec {1:100} [11, 12], Raleigh, NC, USA), cleaved caspase 3 (Cell Signaling Technology {1:50}, Danvers, MA, USA), GFAP (Abcam {1:500} Cambridge, MA, USA), Iba1 (Wako {1:400}, Richmond, VA, USA), CNPase (Abcam {1:200} Cambridge, MA, USA), NeuN (EDM Millipore {1:250} Billerica, MA, USA), Olig2 (Santa Cruz Biotechnology{1:50} Dallas, TX, USA), and secondary antibodies (Species specific Cy3 and FITC 1:125 Jackson Immunoresearch, Westgroove, PA, USA). *N* = 5 animals/group.

### Immunostaining analysis

Digital images obtained from Confocal software were exported to ImageJ. The fluorescence intensity associated with each pixel was determined in sections 750 *×* 750 μm that included 4 sections per animal and 5 animals per group. Excitation and acquisition parameters were adjusted to fully eliminate pixel saturation*,* and all images were collected under identical settings. Cell counting was performed on 4 sections per animal (750 *×* 750 μm each) and 5 animals per group.

### Inflammatory cytokines assay

Assay of proinflammatory cytokines IL-1β, IL-6, and TNF-α was done on the periventricular brain area on postnatal day 6 using Quantikine ELISA kits (R&DSystems) which was used according to the manufacturer’s instructions. *N* = 8 animals/group.

### MRI instrumentation and data acquisition

MRI data were obtained on P15 using 9.4/30 BioSpect Spectrometer (Bruker BioSpin Corp., Germany) equipped with a 72-mm volume coil as a transmitter and a 4-channel mouse brain coil. High-resolution RARE T2-weighted images in axial and coronal plain were acquired with following parameters: TR/TE = 3524/36 ms, FOV = 1.5 cm, matrix = 256 *×* 256, 0.5 mm slice thickness, Nslices = 30, Navg = 4, and RARE Factor = 8.

The animals were anesthetized on P15 using 1–2% isoflurane with carbogen (95%O2+5%CO2). Their respiration and temperature were monitored during the entire course of the experiment using Small Animal Instruments monitoring system (Small Animals Instruments, Inc., Stony Brook, NY, USA).

### MRI data analysis

The axially acquired T2-weighted images (0.5 mm slice thickness) were used for data analysis. Mask of the mouse brain and ventricles were obtained by manually tracing area of interest. Coronally acquired images were used for a conformation. Images were imported into Analyze 7.5 software (Biomedical Imaging Resource, Mayo Clinic, Rochester, MN) for manual and semi-automated volume rendering. We estimated brain volumes for the 0.5-mm gap using Analyze. Total brain tissue volume was defined as the total intracranial volume minus all cerebrospinal fluid (CSF) spaces. *N* = 10 animals/group.

### Neurobehavioral testing and long-term outcome

Forelimb and hindlimb grip strength were measured using a grip strength meter (Columbus Instruments) at 60 days of age. Each session consisted of the average of three tests per animal. Rotarod device (Columbus Instruments, Columbus, OH, USA) was used to measure motor function and balance at P60. Each session consisted of the average of three trials on the elevated accelerating rotarod beginning at 5 RPM, measuring the time the mouse was able to remain on the rod. At P60, animals were tested in an open field analysis (San Diego Instruments, San Diego, CA, USA). Animals were given several minutes to adapt to the testing chamber before the beginning of testing. Open field data was digitally recorded for 30 min and subsequently analyzed by Noldus Ethovision tracking software [13]. Beam breaks were recorded in the *x*, *y*, and *z* planes and averaged across groups. *N* = 25 animals/group.

### Statistical analyses

For all statistical tests, Graph Pad Prism 7 software (La Jolla) was used. Statistical analysis of mean differences between groups was performed by using one-way ANOVA, followed by a Bonferroni post-hoc analysis. All *p* values and *n* values are indicated in figure legends. No sex difference was found in the studies.

## Results

### CSF-1R-cre is expressed mainly in microglia in our mouse model

To determine the relevance of NF-κB inhibition in myeloid cells mainly microglia in PVL pathology, we crossed mice with conditional mutant of IKKb (IKKb^f/f^), which have exon 3 of the ikbkb (IKKb) gene flanked by loxP sites to a strain expressing cre recombinase driven by the promoter for the gene c-fms, which encodes colony stimulating factor receptor 1 (CSF-1R). CSF-1R is expressed throughout the mononuclear phagocyte system of the mouse, including monocyte-derived macrophages, although mainly microglia express CSF-1R in the postnatal mouse brain [14]. Since homozygous mice devoid of IKKb in myeloid cells display severe immune dysfunction, we evaluated heterozygous mice. Heterozygous deletion of IKKb in myeloid cells results in approximately a 50% reduction in NF-κB activity in the CNS.

**Fig. 1:**
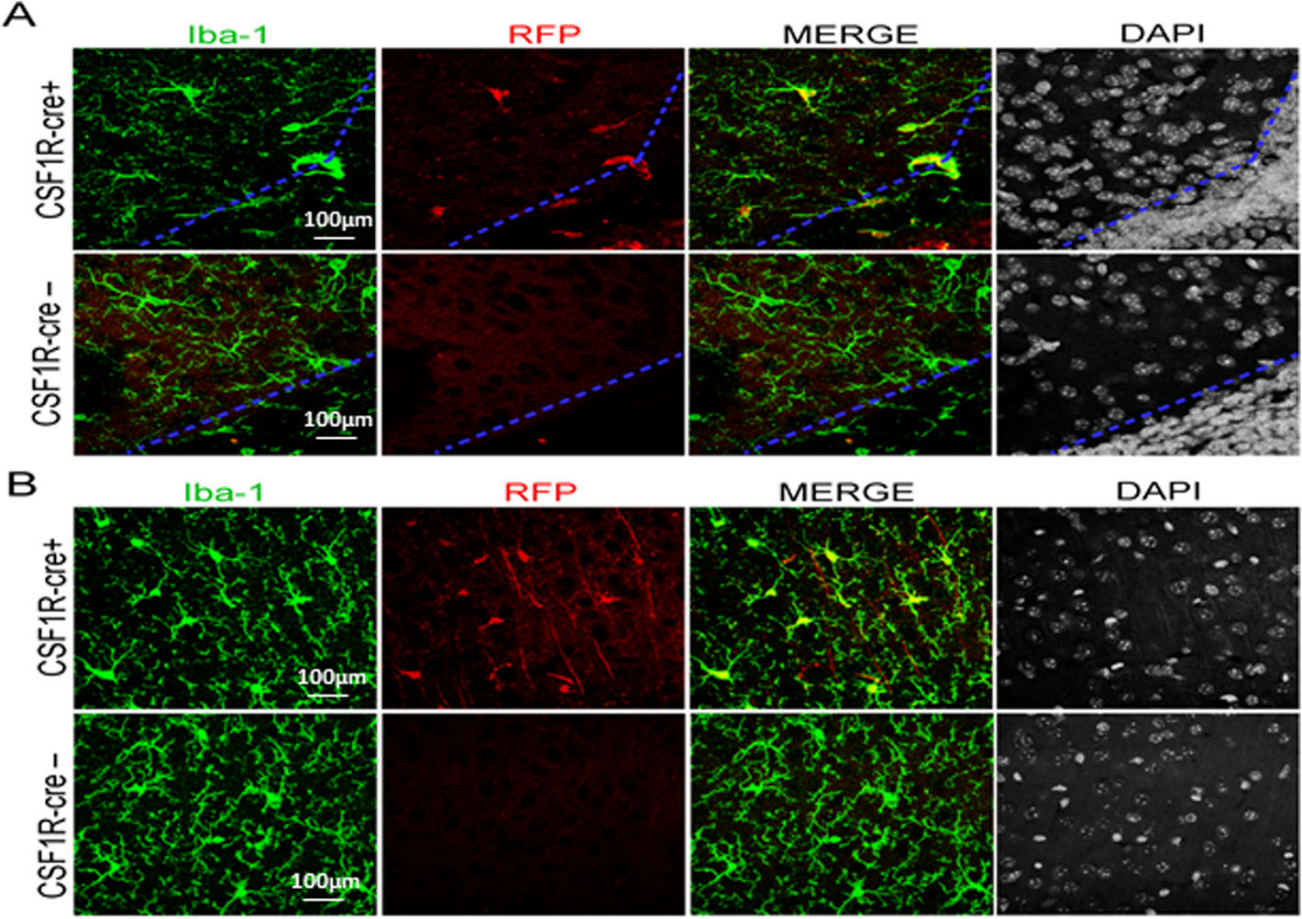
CSF-1R-cre is selectively expressed in microglia in the CNS. CSF-1R is expressed in microglia. CSF-1R-cre mice was crossed to a Rosa26 line that expresses tdTomato (RFP) confirms microglial Iba1 expression in all cre-expressing cells. **a** Robust RFP expression in Iba1+ microglia throughout the neonatal mouse brain including microglia in the periventricular area. **b** RFP expression in Iba1 microglia in the cortex. This proves that the cre expression is restricted to microglia

To confirm that cre expression was restricted to Iba1-expressing microglia in the brain, we crossed CSF-1R-cre mice to a Rosa26 line that expresses tdTomato (RFP) in all cre-expressing cells. We observed robust RFP expression in Iba1+ microglia throughout the neonatal mouse brain including microglia near the lateral ventricles (Fig. 1a) and in the cortex (Fig. 1b). This proves that cre expression is restricted to microglia.

Since there is no activation of NF-κB without hypoxia ischemia, sham room air (RA) Cre + and sham (RA) Cre – showed the same results in all histopathology and molecular testing. Therefore, through the experiments, one RA group was displayed.

Neonate mice were subjected to hypoxia ischemia insult on P5 by temporary bilateral carotid artery ligation followed by transfer to hypoxia chamber.

Three mice groups were studied. RA or Sham group, CSF-1R cre – IKKβ^flox/wt^ mice subjected to HI (HI Cre–), and CSF-1R cre + IKKβ^flox/wt^ mice subjected to HI (HI Cre +).

### NF-κB inhibition in microglia leads to white matter volume preservation

**Fig. 2:**
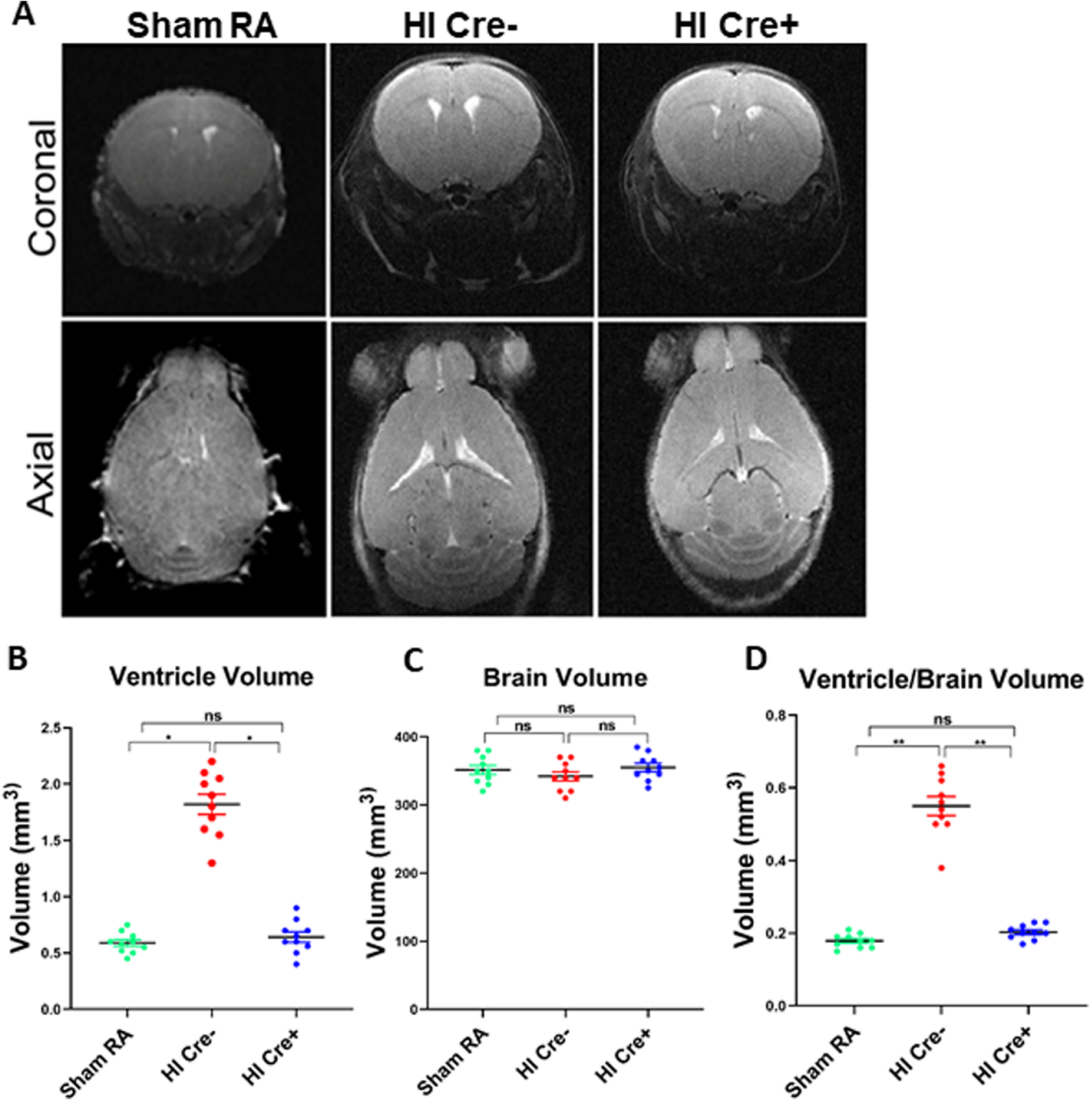
NF-KB inhibition in microglia leads to white matter volume preservation. MRI volumetric analysis at postnatal day 15 comparing sham RA, HI IKKβ^flox/wt^ CSF-1R Cre− (HI Cre−), and HI IKKβ^flox/wt^ CSF-1R Cre+ (HI Cre+), using the BioSpec 94/30 imaging system is a 9.4 T horizontal bore magnet operates at 400 MHz and runs ParaVision™4.0 software. **a** Coronal (top) and axial (bottom) brain slices. **b** The two lateral ventricle volumes measured by paravision software. **c** Total brain volume includes brain volume + lateral ventricle volume measured by paravision software. **d** The ratio of both lateral ventricle volumes to total brain volume measured by paravision software. *N* = 10 animals/group. Scatter dot plot showing mean ± SE. indicates*P < 0.05. **P ≤ 0.01

MRI volumetric analysis at P15 (10 days post-HI), using the BioSpec 94/30 Imaging 9.4 T, detected enlarged ventricles in mice subjected to hypoxia ischemia (Fig. 2a). We observed a significant decrease of lateral ventricle volume in HI Cre+ as compared to HI Cre− mice (*P* < 0.05) (Fig. 2b). There was no significant difference in total brain volume between the three groups (Fig. 2c). The ratio of ventricle volume to total brain volume was significantly decreased in the HI Cre+ in comparison to that in the HI Cre− group (*P* < 0.05) (Fig. 2d). Less ventriculomegaly indicates white matter volume preservation in HI Cre+.

### NF-κB inhibition in microglia decreases ventriculomegaly and neuronal cell death in HI model

**Fig. 3:**
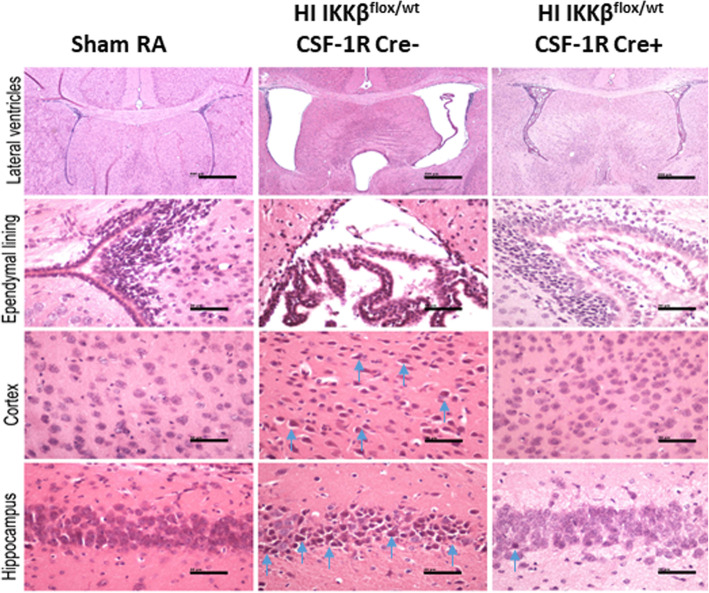
NF-κB inhibition in microglia decreases ventriculomegaly and neuronal cell death in PVL model. Representative H&E coronal brain sections of lateral ventricle, ependymal lining, cortex, and hippocampus of the studied groups, sham RA, HI IKKβ^flox/wt^ CSF-1R Cre− (HI Cre−), and HI IKKβ^flox/wt^ CSF-1R Cre+ (HI Cre+) at postnatal day 15. Blue arrows point to neuronal cell death. Scale bar 10 μm except for lateral ventricle panel is 40 μm. *N* = 12 animals/group

The HI Cre− group had markedly enlarged lateral ventricles and third ventricle shown by hematoxylin and eosin staining, while HI Cre+ only had minimal increase in lateral and third ventricle size comparable to RA controls. Ventricle enlargement which is caused by white matter loss after HI was strikingly reduced in the HI Cre + group and resembled non-injured controls (Fig. 3). HI Cre+ show reduced neuronal damage in cortex and hippocampus compared to the HI Cre− group. Ependymal lining was thickened and had increased cellular mitosis, indicating injury in the HI Cre− group compared to the HI Cre+ group. These findings indicate that prophylactic inhibition of NF-κB in the microglia decreased neuronal damage and oligodendroglial cell loss (Fig. 3).

### NF-κB inhibition in microglia rescues oligodendroglia in HI model

**Fig. 4:**
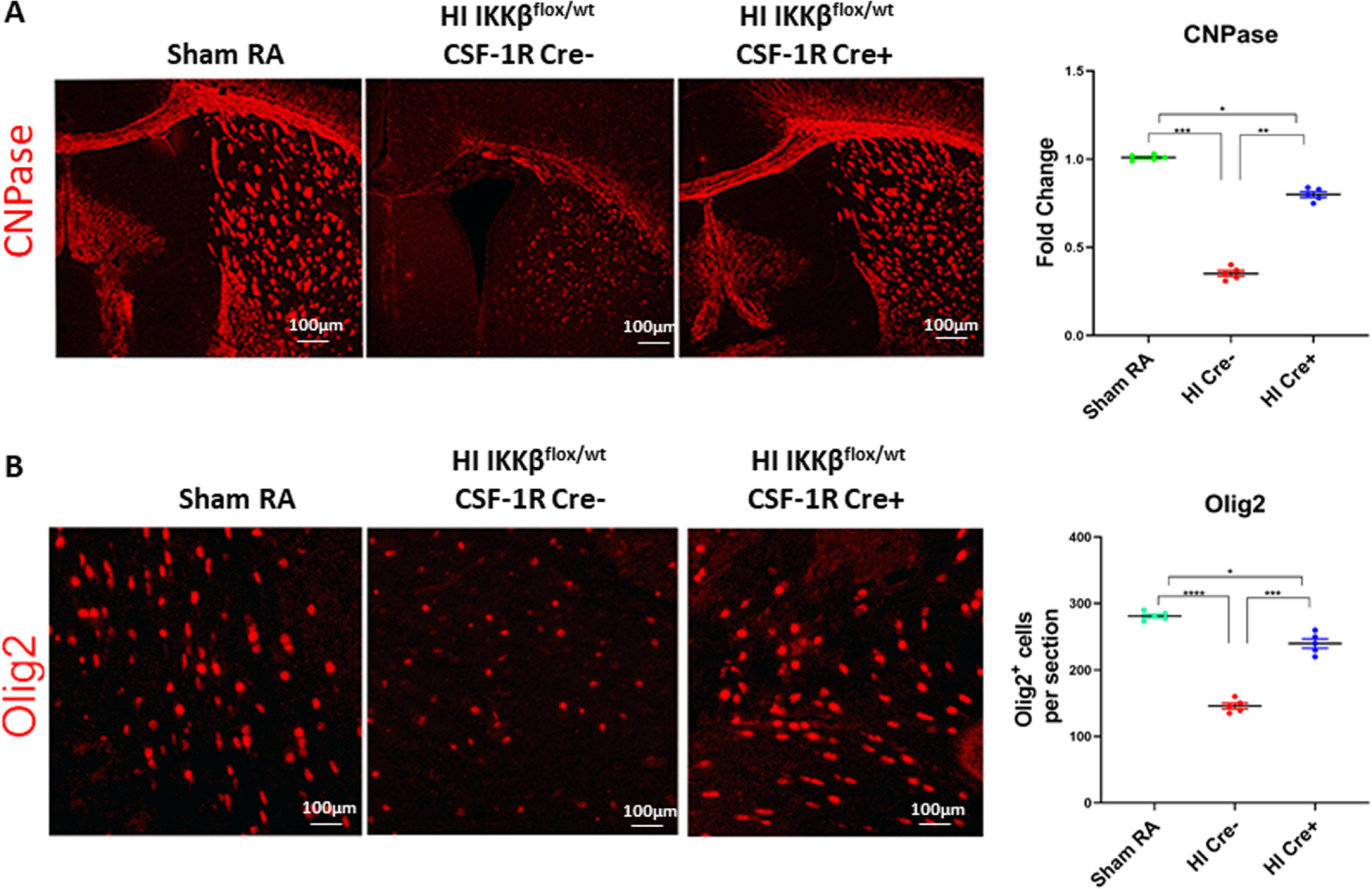
NF-KB inhibition in microglia rescues oligodendroglia in HI model. **a** CNPase in red (early oligodendroglia differentiation marker) in the periventricular area of RA, HI IKKβ^flox/wt^ CSF-1R Cre− (HI Cre−), and HI IKKβ^flox/wt^ CSF-1R Cre+ (HI Cre+) at postnatal day 15. Quantification of CNPase intensity as fold change where sham = 1. **b** Olig 2 in red (early oligodendroglia transcription factor) in the periventricular area of RA, HI IKKβ^flox/wt^ CSF-1R Cre− (HI Cre−), and HI IKKβ^flox/wt^ CSF-1R Cre+ (HI Cre+) at postnatal day 15. Quantification of Olig2-positive cells per section. Scale bar = 100 μm *N* = 5 animals/ group. Four sections/animal. Scatter dot plot showing mean ± SE. *P < 0.05. **P ≤ 0.01. ***P ≤ 0.001, ****P ≤ 0.0001

To provide further evidence that decreased ventriculomegaly is due to myelin preservation, we stained for 2′,3′-cyclic-nucleotide 3′-phosphodiesterase (CNPase). Levels of CNPase were significantly decreased in the HI Cre− group as compared to those in the RA control (Fig. 4a). There was only a mild decreased in CNPase in the HI Cre+ group indicating oligodendrocyte differentiation and white matter preservation (Fig. 4a). Similar results were shown using Oligodendrocyte transcription factor (Olig 2), an oligodendrocyte precursor (Fig. 4b). Thus, prophylactic inhibition of NF-κB in the microglia increase oligodendroglial precursors and promotes differentiation.

### NF-κB modulates microglial activation to an inflammatory, neurotoxic phenotype

**Fig. 5:**
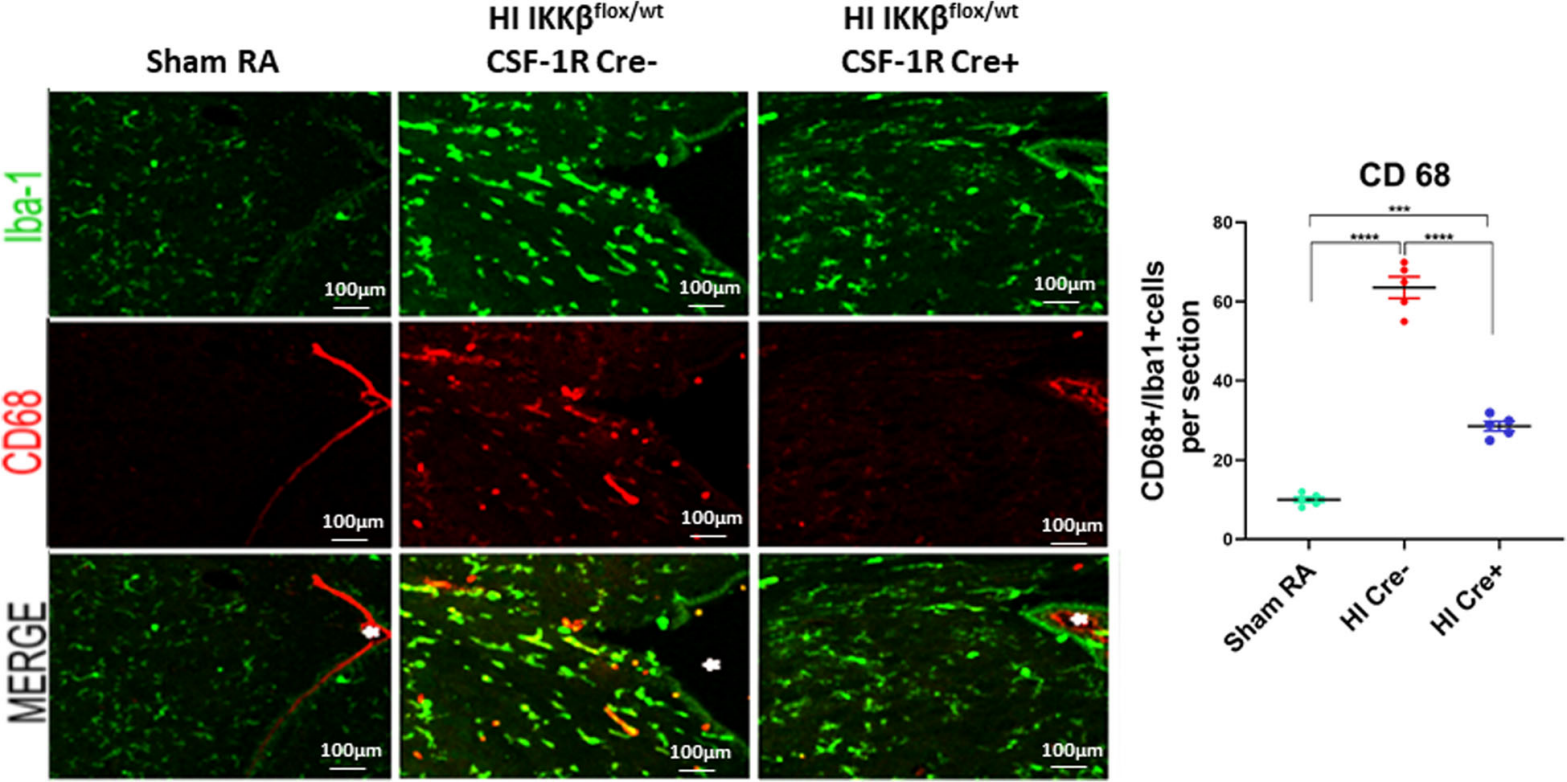
NF-κB modulates microglial activation to an inflammatory, neurotoxic phenotype. **a** Immunostaining for all microglia (Iba1) in green (top) and CD 68 (marker of activated microglia M1 only) in red (middle). Co-localization in yellow indicating amount of activated microglia at postnatal day 15. * indicates lateral ventricle. Quantification of CD68/Iba1 per section. Scale bar = 100 μm *N* = 5 animals/group and 4 sections/animal. Scatter dot plot showing mean ± SE. ***P ≤ 0.001, ****P ≤ 0.0001

We hypothesized that the sparing of white matter in HI Cre+ pups was due to decreasing the proinflammatory microglia response. Therefore, we evaluated HI Cre− and HI Cre+ pups for CD68, a marker for activated proinflammatory microglia. Microglia in mice subjected to HI exhibit an activated proinflammatory, CD68+ phenotype. HI Cre+ mice displayed reduced CD68+ microglia in the periventricular white matter compared to the HI Cre− mice (Fig. 5a).

### NF-κB inhibition in microglia decreases proinflammatory milieu

**Fig. 6:**
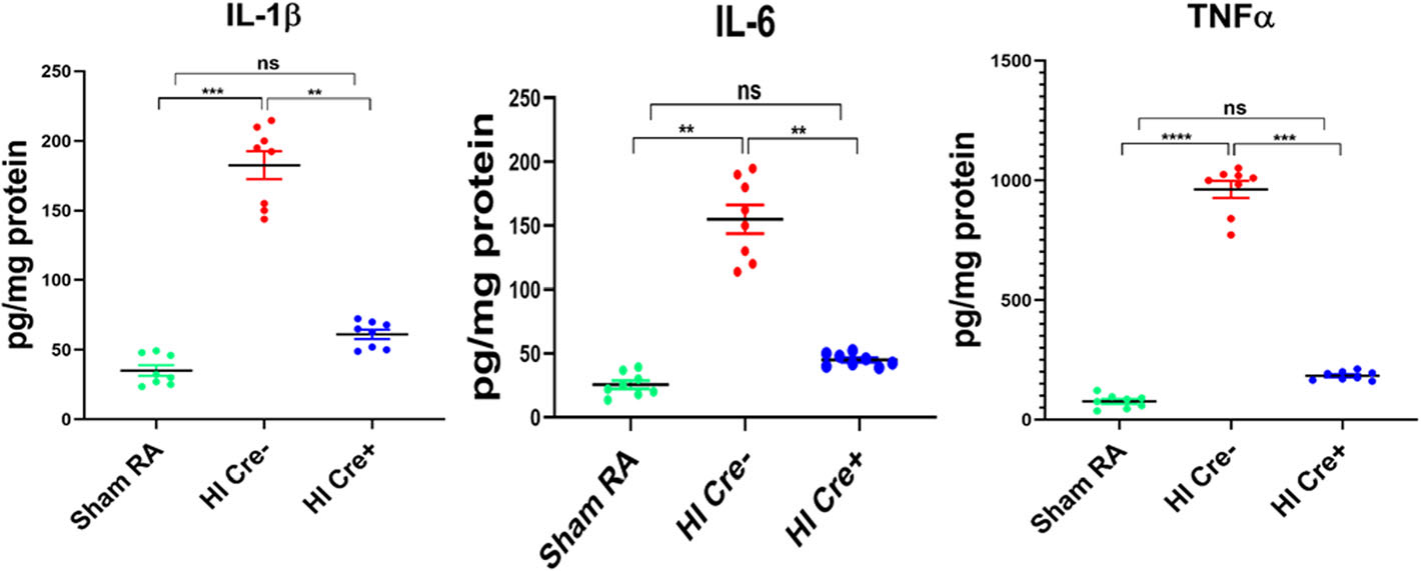
NF-KB inhibition in microglia decreases proinflammatory milieu. ELISA assay of proinflammatory cytokines IL-1β, IL 6, and TNF-α from brain homogenates at postnatal day 6. These cytokines are known to peak in both human and animal PVL models HI. *N* = 8 animals/ group. Scatter dot plot showing mean ± SE. **P ≤ 0.01. ***P ≤ 0.001, ****P ≤ 0.0001

Additionally, proinflammatory mediators IL1β, IL6, and TNF-α, which are increased in human PVL, were also significantly increased in HI Cre− mice as compared to HI Cre+ mice (*P* < 0.05) (Fig. 6). Collectively, these data demonstrate reducing NF-κB activity in microglia dampened proinflammatory microglial activation in a mouse model of PVL.

### NF-κB activation increases nitrative stress, causing injury to the vulnerable oligodendroglia

**Fig. 7:**
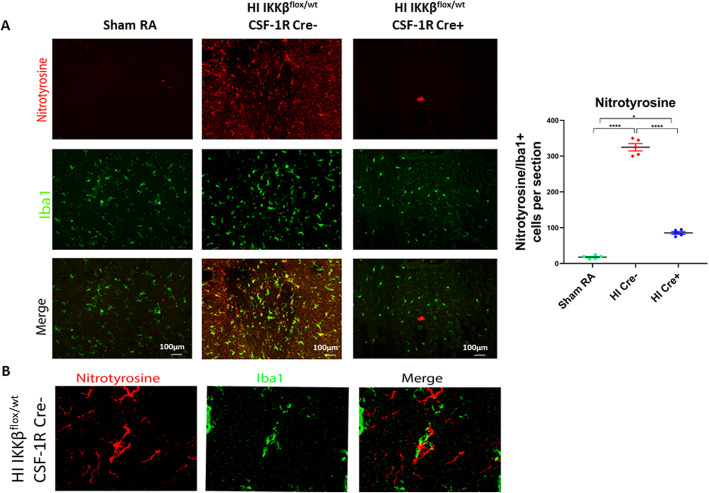
NF-κB activation increases nitrative stress, causing injury to the vulnerable oligodendroglia. **a** Immunohistochemistry of coronal sections of the periventricular brain area of postnatal day 15 pups. Top panel shows Nitrotyrosine (marker of nitrative stress) in red. Middle panel shows Iba1 (microglial marker) in green. Lower panel shows Co-localization in yellow nitrotyrosine secreted by microglia. Quantification of nitrotyrosine/Iba1 per section. Scale bar = 200 μm. *N* = 5 animals/group and 4 sections/animal. Scatter dot plot showing mean ± SE. *P < 0.05, ****P < 0.0001. **b** Higher magnification in the HI IKKβ^flox/wt^ CSF-1R Cre− (HI Cre−) group showing the co-localization of nitrotyrosine to Iba1 indicating that microglia is source of the nitrative stress (nitortyrosine)

Free radical injury to the developing oligodendroglial cells (OL) underlies the pathogenesis of PVL and the hypomyelination seen in long-term survivors. In human PVL, free radical injury leads to increased lipid peroxidation and nitrotyrosine production. Nitrotyrosine is secreted by microglia as shown by the co-localization of nitrotyrosine with Iba-1. Nitrotyrosine expression is significantly increased in HI Cre− as compared to HI Cre+ (*P* < 0.05) (Fig. 7). Pre-oligodendrocytes are the most vulnerable cells to nitrative stress; therefore, they undergo cell loss leading to ventriculomegaly. HI Cre+ group exhibits reduction in nitrative stress as a result of decrease nitrotyrosine release from microglia. This leads to a decrease in pre-oligodendroglial loss in the HI Cre+ as compared to the HI Cre− group.

### Improved long-term neurobehavioral outcome with microglial inhibition of NF-κB

**Fig. 8:**
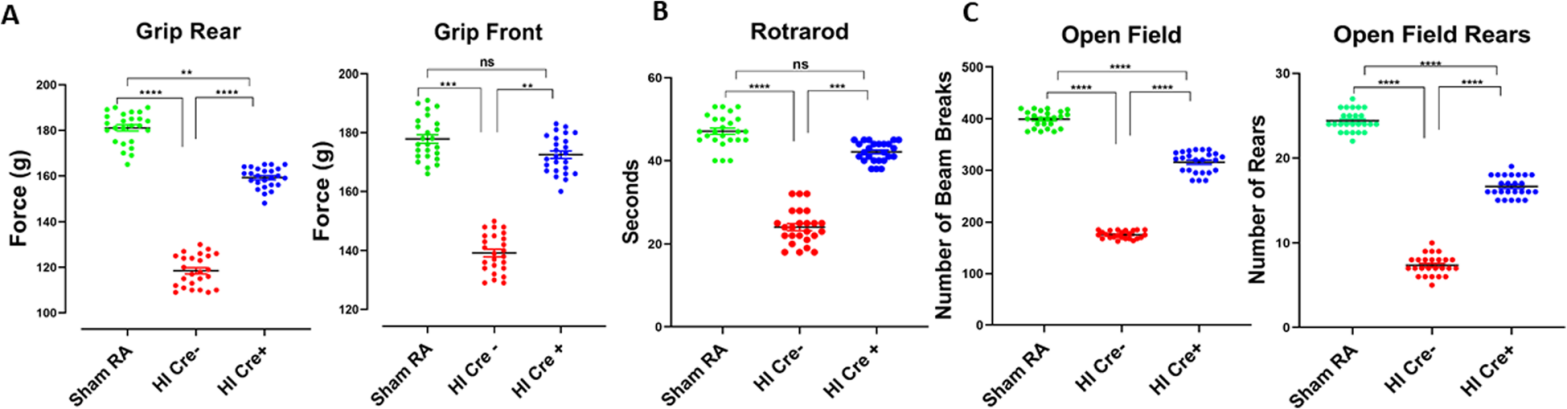
Improved long-term neurobehavioral outcome with microglial inhibition of NF-KB. **a** Testing for rear and front grip strength at postnatal day 60 (P60). **b** Testing of latency to fall in seconds by rotarod for coordination assessment at P60. **c** Number of beam breaks and number of rears in the open field test as an assessment of motor function at P60. *N* = 25 animals/group. Scatter dot plot showing mean ± SE. **P ≤ 0.01, ***P ≤ 0.001, ****P ≤ 0.0001

To investigate if the improvement in histopathological findings and oligodendroglial survival along with a reduction in inflammation translate to improvement in neurodevelopmental outcome in HI Cre+ compared to HI Cre− group, neurobehavioral analysis was performed at P60. Rear grip strength was significantly higher in HI Cre+ compared to HI Cre− group mice (Fig. 8a). Front grip strength was also improved in HI Cre+ versus HI Cre− group (Fig. 8a). On rotarod, HI Cre− group had a more tendency to fall indicating impaired coordination in HI Cre− as compared to HI Cre+ group mice (Fig. 8b). By analyzing open field, number of rears and beam breaks were also significantly decreased, indicating worsening locomotion in HI Cre− versus HI Cre+ group mice (Fig. 8c). These long-term findings mimic human PVL neurodevelopmental outcomes such as diplegia/paresis of the lower limbs, incoordination, and tendency to fall along with reduced activity and attention.

## Discussion

Periventricular leukomalacia (PVL) is a major neuropathologic brain injury and is the most common cause of cerebral palsy (CP) in premature infants. Currently, there is no available treatment for this devastating injury. Microglia are one of the main inflammatory cells involved in the pathogenesis of PVL. Here, we show that NF-κb activation in microglia is playing a major role in the pathogenesis of hypoxic ischemic injury of the immature brain. Heterozygous inhibition of NF-κB in microglia offers significant protection after exposure to HI insult. These findings suggest specific inhibition of microglial NF-κb could be a novel target in the prophylaxis and treatment of HI injury of the immature brain.

Phenotypically, this HI mouse model mimics human PVL in the form of hind limb paresis and incoordination. It displays great similarity to premature infants with severe PVL who exhibit diplegia, incoordination, and CP [10]. Also, this model mimics human PVL histologically in the form of white matter loss leading to ventriculomegaly, with oligodendroglial cell loss and maturational arrest [10].

During HI insult, an inflammatory response cascade is activated, which leads to massive cell damage and necrosis. Hypothermia or hyperthermia can alter this inflammatory response and thus the degree of HI injury. In our study, temperature was measured during surgery and in the hypoxia chamber and was maintained at 36.5 °C in both HI groups. Pups were returned to their dams, and no further measurements were obtained due to technical difficulties given the small size of the pups (2–3 g). Dams keep pups warm and keep them normothermic, but there is a potential that pups became hypothermia after HI which could potentially alter the degree of HI injury. Recent human studies support contributory role for proinflammatory cytokines in the pathogenesis of PVL [15]. Incidence of PVL and CP in premature infants is increased in the presence of maternal, placental, or fetal infection [16–27]. Elevated levels of IL-6 in the cord blood [3, 28], elevated levels of IL-6 and IL-1β in amniotic fluid [4, 29], and elevated levels of all interferons and IL-1 and IL-6, among other cytokines, in neonatal blood have all been associated with increased incidence of PVL and CP in premature infants [19, 30, 31]. In animal models, IL-1β, IL-6, MIP, IL-9, and TNF-α seem to play an important role [15, 32, 33]. Cytokines and bacterial products can cause direct injury to the developing OLs. It was shown that TNF-α is toxic to OLs [11, 34–40]; others demonstrated that OLs show high toxicity by interferon-y. Ιmmature OLs in a culture are more vulnerable to the cytotoxicity of interferon-y than are mature OLs [6, 36, 41, 42]. In addition, TNF-α potentiates this toxicity of interferon-y to developing OLs [39].

Anti-inflammatory treatments may represent a useful strategy in the treatment of PVL, where clinical conditions would favor a post-insult treatment strategy. Minocycline, as a microglial in-activator, showed protective effect when administered following HI insult [5]. Attenuation of inflammatory reaction by pharmacological inhibition of NF-κB significantly reduced brain injury after HI insult as shown by improvement in long-term motor and cognitive functions [8, 9]. In our study, we explored both the dynamic and cell-specific activation of NF-κB during HI insult and found that specific inhibition of NF-κB in microglia in neonate mice exposed to HI insult leads to decrease microglia activation. A decrease in microglial activation led to less oligodendroglial destruction (Fig. 4).

It is well established in animal models that ischemia reperfusion is accompanied rapidly by activation of microglia, secretion of cytokines, and mobilization, adhesion, and migration of macrophages and inflammatory cells [43, 44]. While microglia are the main cells in the brain that express CSF-1R, monocytes and macrophages also express CSF-1R and can be seen in the brain in HI due to increased blood brain barrier permeability. Therefore, it is likely that part of this neuroprotective effect is due to inhibition of NF-κB in infiltrating monocytes and macrophages. Whether induced by infection or ischemia, these inflammatory responses could be detrimental to developing OLs [35, 45, 46]. Vasoactive effects of certain cytokines and nitrogen species released, as part of the inflammatory cascade, can impact cerebrovascular regulation, impair perfusion, and thereby increase the risk for ischemic injury [47, 48]. In HI Cre+ mice, there was a significant reduction of inflammatory cytokines within 24 h post-HI insult as compared to HI Cre− mice (Fig. 6).

Diffuse oligodendroglial (OL) injury in PVL is related to moderate ischemia. Early differentiating OL or pre-OL is vulnerable to free radical attack, whereas the mature OL is resistant [49, 50]. Increase accumulation of free radicals in OL precursors, with limited and deficient antioxidants defense, leads to hydrogen peroxide accumulation, which produces the deadly hydroxyl radical [51–53]. In human PVL, free radical injury is supported by evidence of oxidative and nitrative stress with markers to lipid peroxidation and nitrotyrosine [2]. Free radicals are both a cause and a result of inflammation. Reperfusion of ischemic tissues is associated with microvascular injury, particularly due to increased permeability of capillaries and arterioles, which leads to an increase of diffusion and fluid filtration across the tissues. These activated injured endothelial cells produce more reactive oxygen and nitrogen species which trigger more inflammatory response. NF-κB can also induce nitric oxide synthase in glial cells resulting in the production of nitric oxide (NO) and related neurotoxic reactive oxygen species [54]. NO itself can induce pre-OL damage by two mechanisms: one involving the direct effect of nitric oxide on pre-OL mitochondrial integrity and function, and the other involving an activation of microglia and subsequent release of reactive nitrogen species [12]. Activated microglia release NO and nitrogen species, which mediate neurotoxicity in several neurodegenerative diseases and in HI insults [55, 56]. In our model, inhibition of NF-κB in microglia, not only attenuates the inflammatory response elicited by cell injury and free radical accumulation, but also modulates NO release. Our results showed a significant reduction of activated microglia in HI Cre+ mice, as one of the main sources of released NO and nitrotyrosine (Figs. 5 and 7).

In our HI model, long-term neurobehavioral evaluation showed a significant decrease in rear grip strength, overall locomotion, and incoordination mimicking human PVL who present with diplegia and incoordination (Fig. 8). These findings can be explained by periventricular white matter loss as evident in our MRI studies showing significantly increased ventriculomegaly in HI group assessed by ventricle volume as well as by calculating the ventricle/brain volume ratio (Fig. 2). The ventricle volume was statistically increased in HI Cre− group at 2.5 times the volume of the HI Cre+ group. The HI Cre− group had slightly smaller total brain volume than the HI Cre + group but this was not statistically significant. Ventriculomegaly and white matter loss are also shown in our histopathological studies (Fig. 3). Severity of motor impairment and cognitive impairment were found to be closely associated with the lateral ventricular volumes [57]. Reduction of the periventricular white matter and the degree of lateral ventricle expansion are the main causes of dysfunctions and damage of vision and hearing as well as intellectual impairment and CP in children with PVL [58, 59]. Specific inhibition of NF-κB in microglia in neonate mice exposed to HI insult led to a significant attenuation of ventriculomegaly as shown by measuring both ventricle volumes. These MRI Findings, in addition to the decreased neuronal damage and oligodendroglial loss, shown in our histopathological studies, explain the significant improvement in neurodevelopmental long-term outcome among HI Cre+ group.

NF-κB is a key transcription factor involved in the regulation of cytokine production [60]. NF-κB can modulate the expression of apoptosis-promoting cytokines such as TNF-α and FAS ligand (FASL) [61]. In models of ischemia, NF-κB activation appears to contribute to brain damage and mice lacking the p50 subunit of NF-κB demonstrate decreased infarct volumes [62, 63]. Many studies highlight the pivotal role of activation of NF-κB in glial cells that lead to production of neurotoxins [64]. In a trial to attenuate the proinflammatory reaction of astroglia mediated by NF-κB expression, transgenic models with relatively inhibited NF-κB expression were used and showed a significant reduction in white matter injury in disease models of autoimmune encephalomyelitis and ischemic stroke [8]. Other studies used certain small cell-penetrating peptides (CPPs) to inhibit NF-κB activation in animal models of different disorders, e.g., diabetes mellitus type 1 [65], inflammation (acute and chronic) [12, 66–68], and cancer [69, 70]. Most of CCPs studies (in vitro and in vivo studies) showed significantly attenuated inflammatory infiltration, decreased cell necrosis, and degeneration with resultant amelioration of disease severity [71]. Our study as well as others suggests that focusing on NF-κB activation is a realistic target for the development of therapeutic approaches.

## Conclusion

NF-κB regulates a large number of essential cellular activities and is essential for many developmental aspects. This precludes “blanket” inhibition of NF-κB as a clinical intervention. NF-κB activation in glutaminergic neurons is involved in learning and memory, dendritic arborization, and axonal outgrowth [72]. When targeting NF-κB in the brain, cell-type specificity is required so the functional benefits are achieved by dampening microglial activation and inflammation. Our study highlights the fact that NF-κB activation in microglia is a major contributor of white matter injury in the premature brain. Using a specific inhibitor of microglial NF-κB can be a new prophylactic/therapeutic approach for hypoxic ischemic brain injury.

## Data Availability

The datasets used and/or analyzed during the current study are available from the corresponding author on reasonable request.

## Abbreviations

PVL: Periventricular lekomalacia
CP: Cerebral palsy
HI: Hypoxia ischemia
CSF-1R Cre +: Colony stimulating factor receptor 1-Cre positive
RFP: Red fluorescent protein
CNPase: Cyclic-nucleotide 3′-phosphodiesterase
Olig 2: Oligodendrocyte transcription factor
IL-1β: Interleukin 1β
IL-6: Interleukin 6
TNF-α: Tumor necrosis factor-α
OL: Oligodendroglial cells
NO: Nitric oxide
FASL: FAS ligand
CPPs: Small cell-penetrating peptides
